# Association between arsenic metabolism gene polymorphisms and arsenic-induced skin lesions in individuals exposed to high-dose inorganic arsenic in northwest China

**DOI:** 10.1038/s41598-017-18925-3

**Published:** 2018-01-11

**Authors:** Lanrong Luo, Yuanyuan Li, Yanhui Gao, Lijun Zhao, Hongqi Feng, Wei Wei, Chuanying Qiu, Qian He, Yanting Zhang, Songbo Fu, Dianjun Sun

**Affiliations:** 1 Center for Endemic Disease Control, Chinese Center for Disease Control and Prevention, Harbin Medical University; Key Lab of Etiology and Epidemiology, Education Bureau of Hei Long Jiang Province & Ministry of Health, Harbin, 150081 China; 2 Dongcheng Haidian District Center for Disease Control and Prevention, Beijing, 100009 China

## Abstract

Individuals in a given environment contaminated with arsenic have different susceptibilities to disease, which may be related to arsenic metabolism, age, gender, genetics and other factors. This study recruited 850 subjects, including 331 cases and 519 controls, from populations exposed to high levels of arsenic in drinking water in northwest China. Genotypes were determined using a custom-by-design 48-Plex SNPscan^TM^ kit. The results indicated that subjects who carried at least one C allele for GSTO1 rs11191979 polymorphism, at least one A allele for GSTO1 rs2164624, at least one A allele for GSTO1 rs4925, the AG genotype for GSTO2 rs156697, the AG genotype or at least one G allele for GSTO2 rs2297235 or the GG genotype or at least one G allele for PNP rs3790064 had an increased risk of arsenic-related skin lesions. In addition, the haplotype CT between rs4925 and rs11191979 appeared to confer a high risk of arsenic-included skin lesions (OR = 1.377, 95% CI = 1.03–1.84), as did the haplotype GCG among rs156697, rs157077 and rs2297235 (OR = 2.197, 95% CI = 1.08–4.44). The results showed that the variants of GSTO1, GSTO2 and PNP render the susceptible toward developing arsenic-induced skin lesions in individuals exposed to high-dose inorganic arsenic in northwest China.

## Introduction

Arsenic contamination is a severe public health problem. More than seventy countries and regions around the world have high arsenic contents, including Bangladesh, China, Mexico, India, and Argentina^1–3^. In arid and semiarid regions in northwest China, individuals develop a typical drinking water-type endemic arsenic-poisoning lesion, and the arsenic content of the groundwater in the area is higher than the 10 μg/L standard set by the World Health Organization^4^. As a potential environmental carcinogen, arsenic can affect health, and long-term exposure to drinking water with high levels of arsenic can increase the risk of cancers, including skin, lung, bladder, kidney and liver cancers, and also influence various non-cancer diseases, including cardiovascular and cerebrovascular diseases, diabetes, reproductive and developmental disorders, and neurological and cognitive dysfunction^5–8^. In addition, arseniasis, also known as chronic arsenic poisoning, can cause different skin manifestations, including palmoplantar keratosis and pigmentation or depigmentation on the chest and back and in severe cases can cause skin cancer or Bowen’s disease^9–11^. Although more than 100 million people are exposed to arsenic worldwide, many studies have shown that only a portion of them exhibit arsenic-induced skin lesions^12,13^. This phenomenon may be caused by genetic differences^14^, as multiple epidemiological studies have revealed that genetic polymorphisms play an important role in susceptibility to arsenic poisoning^9,15,16^.

The pathogenic mechanism of inorganic arsenic is very complex and is most likely multifactorial^17^. Recently, many epidemiological studies have reported that inter-individual variations in arsenic metabolism can result in differences in susceptibility to arsenic-induced skin lesions^18^; such gene polymorphisms influence the proteins encoded and thus affect enzyme structure and function^19,20^. Two possible metabolic pathways for arsenic have been proposed in mammals^5^, with most studies favoring the classical metabolic pathway^21^ in which (1) pentavalent arsenic (As^V^) is reduced to trivalent arsenic (As^III^) and (2) s-adenosyl methionine (SAM) provides a methyl donor for oxidative methylation of trivalent arsenic, with trivalent arsenic producing mono-, di- and trimethylated derivatives^12,22^. Many types of enzymes participate in this metabolic pathway, though arsenic (III) methyltransferase (AS3MT), glutathione *S*-transferase omega-1 (GSTO1) and omega-2 (GSTO2) and purine nucleoside phosphorylase (PNP) are considered the main enzymes^3,19,23^. AS3MT, an S-adenosyl methionine (SAM)-dependent enzyme, is essential for oxidative methylation of trivalent arsenic during the arsenic biotransformation process^20^. GSTO1 and GSTO2 are important rate-limiting enzymes in arsenic metabolism and catalyze reduction of methyl arsenic^24^. Previous studies have shown that PNP reduces As^V^ to As^III^ in the rat liver and calf spleen^25,26^. Genetic polymorphisms in the genes encoding these enzymes may cause individual differences in arsenic biotransformation, leading to different individual sensitivities to arsenic.

To explore the correlation between arsenic-induced skin lesions and polymorphisms in arsenic-associated metabolic enzyme genes, we analyzed genetic polymorphisms of 25 polymorphic loci in the four genes mentioned above in samples collected from populations in areas of northwest China with high levels of arsenic in the drinking water. The arsenic concentration in the drinking water in Gansu Province was found to be 969 μg/L, nearly 100 times higher than the highest standard for arsenic in drinking water according to the recommendations of the World Health Organization (10 μg/L). The aim of this study was to determine the existence of a relationship between single-nucleotide polymorphisms (SNPs) of the above-mentioned arsenic-metabolizing genes and the development of arsenic-induced skin lesions in an arsenic-exposed population in northwest China.

## Results

### Characteristics of the studied populations

The general characteristics of the cases and controls are shown in Table 1. No significant differences were observed among the cases and controls with regard to age, gender, smoking status, drinking status, or ethnicity. There were however significant differences in the years of cigarette smoking between the two groups. Consistent with the total arsenic intake in drinking water, concentrations of arsenic species (total (tAs) and inorganic (iAs) as well as monomethylarsonous acid (MMA) and dimethylarsinous acid (DMA)) detected in urine samples showed a statistically significant difference between cases and controls (P < 0.05). The rs1191439 SNP in AS3MT was found to not be in Hardy-Weinberg equilibrium (HWE) and was therefore eliminated.

**Table 1:** General characteristics and polymorphic genes of cases and controls.

Characteristic/Polymorphism	Controls (n = 519) n (%)	Cases (n = 331) n (%)	t/X^2^	P	Polymorphism	Controls (n = 519) n (%)	Cases (n = 331) n (%)	χ^2^	P
Age [years (mean ± SE)]	53.70 ± 11.39	54.74 ± 14.74	1.09	0.276	rs3740392				
Gender					T	746(71.9)	475(71.8)	0.003	1
Male	225(43.4)	156(47.1)	1.166	0.289	C	292(28.1)	187(28.2)		
Female	294(56.6)	175(52.9)			rs3740393				
Smoking					G	763(73.8)	477(73.6)	0.007	0.955
No	382(73.6)	255(77.0)	0.816	0.373	C	271(26.2)	171(26.4)		
Yes	135(26.4)	77(24.0)			rs7085104				
Years of cigarette smoking					A	557(53.7)	355(53.6)	0	1
0	382(73.6)	255(77.0)	7.822	**0**.**020**	G	481(46.3)	307(46.4)		
1–35	109(21.0)	48(14.5)			rs7085854				
>35	28(5.4)	28(8.5)			T	845(81.4)	544(82.2)	0.16	0.7
Drinking					C	193(18.6)	118(17.8)		
No	427(82.3)	286(86.4)	2.551	0.126	rs7098825				
Yes	92(17.7)	45(13.6)			T	753(72.8)	469(72.4)	0.04	0.866
Ethnicity					C	281(27.2)	179(27.6)		
Han	499(96.1)	310(93.7)	2.938	0.229	GSTO1/rs11191979				
Hui	12(2.3)	14(4.2)			T	860(82.9)	516(77.9)	6.31	0.013
Tibetan	8(1.5)	7(2.1)			C	178(17.1)	146(22.1)		
Water arsenic concentration*	0.1239 ± 0.0083	0.1245 ± 0.0052	0.06	0.952	rs11509438				
iAs*	38.44 ± 2.676	112.26 ± 7.722	9.03	<0.05	G	1026(99.0)	642(98.5)	1.11	0.365
MMA*	39.89 ± 2.416	131.03 ± 8.750	10.04	<0.05	A	10(1.0)	10(1.5)		
DMA*	173.65 ± 8.917	406.85 ± 27.683	8.02	<0.05	rs2164624				
T-As*	252.05 ± 13.171	650.15 ± 40.194	9.41	<0.05	G	877(84.7)	531(80.7)	4.48	0.039
AS3MT/rs1046778					A	159(15.3)	127(19.3)		
T	572(55.2)	371(56.0)	0.11	0.764	rs2282326				
C	464(44.8)	291(44.0)			A	773(74.5)	462(69.8)	4.46	0.039
rs10748835					C	265(25.5)	200(30.2)		
A	567(54.6)	361(54.5)	0.001	1	rs4925				
G	471(45.4)	301(45.5)			C	860(82.9)	516(78.2)	5.73	0.019
rs10883790					A	178(17.1)	144(21.8)		
A	746(71.9)	474(71.6)	0.01	0.912	GSTO2/rs156697				
C	292(28.1)	188(28.4)			A	768(74.3)	450(69.0)	5.51	0.019
rs11191438					G	266(25.7)	202(31.0)		
C	563(54.6)	351(54.2)	0.02	0.88	rs157077				
G	469(45.5)	297(45.8)			T	595(57.3)	371(56.0)	0.27	0.616
rs1191439					C	443(42.7)	291(44.0)		
T	1019(98.2)	646(97.6)	0.69	0.484	rs2297235				
C	19(1.8)	16(2.4)			A	861(82.9)	516(77.9)	6.57	0.011
rs1191442					G	177(17.1)	146(22.1)		
T	747(72.0)	473(71.7)	0.02	0.912	PNP/rs1713420				
A	291(28.0)	187(28.3)			A	920(88.6)	583(88.1)	0.13	0.756
rs1191454					G	118(11.4)	79(11.9)		
A	763(73.5)	489(73.9)	0.03	0.91	rs1760940				
G	275(26.5)	173(26.1)			A	922 (88.8)	577(87.2)	1.07	0.317
rs12416687					C	116(11.2)	85(12.8)		
T	912(87.9)	578(87.3)	0.11	0.763	rs3790064				
C	126(12.1)	84(12.7)			A	943(91.2)	568(87.1)	7.16	0.009
rs3740390					G	91(8.8)	84(12.9)		
C	762(73.4)	489(69.3)	3.56	0.065					
T	276(26.6)	217(30.7)							

### Screening for gene polymorphisms and analyzing allele frequencies

Information for all genes was obtained from an SNP database (https://www.ncbi.nlm.nih.gov/snp/?term), and we chose the higher allele frequencies of SNP in the Chinese population. Only the mutant allele frequency of rs11509438 was less than 10%, whereas the mutant allele frequencies of the other SNPs ranged from 0.1038 to 0.4635. The allele frequencies for GSTO1, GSTO2, AS3MT and PNP among the cases and controls showed a significant difference for GSTO1 SNPs rs11191979, rs2164624, rs2282326 and rs4925, GSTO2 SNPs rs156697 and rs2297235 and the PNP SNP rs3790064 (P < 0.05). In contrast, the allele frequencies of 13 SNPs at the AS3MT locus were not significantly different between the cases and controls.

### Association between gene polymorphisms and the risk of arsenic-induced skin lesions

The main effects of each genotype on skin lesions after adjustment for age, gender, smoking status, drinking status and ethnicity are shown in Table 2. In addition to the analyis resported above, integral analysis of genotypes showed that AS3MT rs11191439 was not in HWE; another 13 SNPs in AS3MT showed no robust association with the risk of arsenic-induced skin lesions. However, each genotype for the six SNPs in GSTO1, GSTO1 and PNP were significantly different between the cases and controls. For GSTO1, individuals carrying at least one C allele for the rs11191979 polymorphism, at least one A allele or the AA genotype for rs2164624 or at least one A allele for rs4925 showed a significant risk of arsenic-induced skin lesions [OR = 1.382 (95% CI, 1.031–1.851) for rs11191979; OR = 1.367 (95% CI, 1.002–1.866) and OR = 1.379 (95% CI, 1.021–1.862) for rs2164624; and OR = 1.350 (95% CI, 1.009–1.808) for rs4925] compared with homozygous wild-type individuals. For GSTO2, subjects who carried the AG genotype for rs156697 and the AG genotype or at least one G allele for rs2297235 had an increased risk of arsenic-induced skin lesions [OR = 1.877 (95% CI, 1.109–3.177) for rs156697 and OR = 2.161 (95% CI, 1.035–4.513) and OR = 1.350 (95% CI, 1.031–1.851) for rs2297235] compared with homozygous wild-type individuals. Compared with homozygous wild-type individuals, those carrying the GG genotype or at least one G allele for rs3790064 of PNP had an increased risk of arsenic-induced skin lesions [OR = 1.468 (95% CI, 1.018–2.118) and OR = 1.520 (95% CI, 1.070–2.158)]. With regard to years of cigarette smoking, individuals who smoked for 1–35 years had an increased risk of arsenic-induced skin lesions [OR = 1.735 (95% CI, 1.152–2.611)] compared with nonsmoking individuals.

**Table 2:** Distribution and risk assessment of genotypes for 24 SNPs between cases and controls.

Gene	Genotype	Controls n(%)	Cases n(%)	P	OR	CI95%		adOR	adCI95%	
AS3MT/rs1046778	TT	163(31.5)	100(30.2)		1.00					
	TC	246(47.5)	171(51.7)	0.597	0.897	0.601	1.341	0.861	0.574	1.293
	CC	109(21.0)	60(18.1)	0.439	1.133	0.826	1.555	1.133	0.822	1.560
	TC + CC	355(68.5)	231(31.5)	0.700	1.061	0.786	1.430	1.046	0.774	1.415
rs10748835	AA	160(30.8)	93(28.1)		1.00					
	AG	247(47.6)	175(52.9)	0.873	0.968	0.648	1.445	0.990	0.661	1.483
	GG	112(21.6)	63(19.0)	0.226	1.219	0.885	1.680	1.247	0.900	1.728
	AG + GG	359(69.2)	238(71.9)	0.396	1.141	0.842	1.545	1.165	0.856	1.585
rs10883790	AA	272(52.4)	170(51.4)		1.00					
	AC	202(38.9)	134(40.5)	0.876	0.960	0.574	1.605	0.926	0.550	1.557
	CC	45(8.7)	27 (8.2)	0.688	1.061	0.794	1.419	1.040	0.776	1.395
	AC + CC	247(47.6)	161(48.6)	0.765	1.043	0.791	1.374	1.019	0.772	1.347
rs11191438	CC	159(30.8)	92(28.4)		1.00					
	CG	245(47.5)	167(51.5)	0.988	1.003	0.673	1.495	1.031	0.689	1.543
	GG	112(21.7)	65(20.1)	0.321	1.178	0.853	1.628	1.216	0.875	1.691
	CG + GG	357(69.2)	232(71.6)	0.456	1.123	0.828	1.524	1.156	0.848	1.577
rs11191442	TT	273(52.6)	170(51.5)		1.00					
	TA	201(38.7)	133(40.3)	0.887	0.964	0.576	1.611	0.928	0.551	1.561
	AA	45(8.7)	27(8.2)	0.683	1.063	0.794	1.421	1.041	0.776	1.397
	TA + AA	246(47.4)	160(48.5)	0.757	1.044	0.792	1.377	1.021	0.772	1.349
rs11191454	AA	283(54.5)	180(54.4)		1.00					
	AG	197(38.0)	129(39.0)	0.672	0.887	0.509	1.545	0.904	0.516	1.584
	GG	39(7.5)	22(6.6)	0.844	1.030	0.770	1.376	1.025	0.765	1.374
	AG + GG	236(45.5)	151(45.6)	0.966	1.006	0.763	1.327	1.005	0.761	1.328
rs12416687	TT	398(76.7)	253(76.4)		1.00					
	TC	116(22.4)	72(21.8)	0.298	1.888	0.570	6.250	1.886	0.567	6.276
	CC	5(1.0)	6(1.8)	0.889	0.976	0.699	1.363	0.954	0.681	1.335
	TC + CC	121(23.3)	78(76.7)	0.933	1.014	0.732	1.404	1.008	0.726	1.399
rs3740390	CC	282(54.3)	180(54.4)		1.00					
	CT	198(38.2)	129(39.0)	0.663	0.884	0.507	1.540	0.905	0.517	1.585
	TT	39(7.5)	22(6.6)	0.890	1.021	0.764	1.364	1.017	0.759	1.363
	CT + TT	237(45.7)	151(45.6)	0.990	0.998	0.757	1.316	0.999	0.756	1.320
rs3740392	TT	272(52.4)	170(51.4)		1.00					
	TC	202(38.9)	135(40.8)	0.767	0.924	0.550	1.554	0.895	0.529	1.514
	CC	45(8.7)	26(7.9)	0.651	1.069	0.800	1.429	1.049	0.783	1.406
	TC + CC	247(47.6)	161(48.6)	0.765	1.043	0.791	1.374	1.021	0.773	1.349
rs3740393	GG	286(55.3)	175(54.0)		1.00					
	GC	191(36.9)	127(39.2)	0.706	0.899	0.517	1.563	0.924	0.528	1.616
	CC	40(7.7)	22(6.8)	0.578	1.087	0.811	1.456	1.079	0.803	1.450
	GC + CC	231(44.7)	149(46.0)	0.711	1.054	0.798	1.393	1.052	0.794	1.394
rs7085104	AA	147(28.3)	87(26.3)		1.00					
	AG	263(50.7)	181(54.7)	0.909	0.977	0.649	1.469	0.964	0.639	1.455
	GG	109(21.0)	63(19.0)	0.364	1.163	0.840	1.611	1.151	0.828	1.599
	AG + GG	372(71.7)	244(73.7)	0.516	1.108	0.813	1.512	1.096	0.801	1.499
rs7085854	TT	343(66.1)	222(67.1)		1.00					
	TC	159(67.1)	100(30.2)	0.633	0.818	0.358	1.867	0.754	0.328	1.737
	CC	17(3.3)	9(2.7)	0.852	0.972	0.719	1.314	0.941	0.693	1.277
	TC + CC	176(33.9)	109(32.9)	0.768	0.957	0.714	1.282	0.922	0.686	1.240
rs7098825	TT	277(53.6)	166(51.2)		1.00					
	TC	199(38.5)	137(42.3)	0.583	0.855	0.488	1.496	0.863	0.490	1.519
	CC	41(7.9)	21(6.5)	0.349	1.149	0.859	1.536	1.138	0.848	1.526
	TC + CC	240(46.4)	158(48.8)	0.508	1.099	0.832	1.451	1.091	0.824	1.444
GSTO1/rs11191979	TT	356(68.6)	202(61.0)		1.00					
	TC	148(28.5)	112(33.8)	0.058	1.997	0.977	4.085	2.027	0.983	4.180
	CC	15(2.9)	17(5.1)	0.060	1.334	0.988	1.800	1.317	0.972	1.785
	TC + CC	163(31.4)	129(39.0)	**0**.**024**	**1**.**395**	**1**.**045**	**1**.**861**	**1**.**382**	**1**.**031**	**1**.**851**
rs11509438	GG	508(98.1)	316(96.9)		1.00					
	GA	10(1.9)	10(3.1)	0.295	1.608	0.662	3.906	1.688	0.687	4.146
rs2164624	GG	372(71.8)	213(64.7)		1.00					
	GA	133(25.7)	105(31.9)	0.351	1.478	0.651	3.357	1.500	0.656	3.428
	AA	13(2.5)	11(3.3)	**0**.**040**	**1**.**379**	**1**.**015**	**1**.**873**	**1**.**367**	**1**.**002**	**1**.**866**
	GA + AA	146(28.2)	116(35.3)	**0**.**030**	**1**.**388**	**1**.**032**	**1**.**866**	**1**.**379**	**1**.**021**	**1**.**862**
rs2282326	AA	289(55.7)	162(48.9)		1.00					
	AC	195(37.6)	138(41.7)	0.085	1.580	0.939	2.658	1.610	0.952	2.721
	CC	35(6.7)	31(9.4)	0.116	1.262	0.944	1.689	1.266	0.943	1.699
	AC + CC	230(44.3)	169(51.1)	0.055	1.311	0.994	1.728	1.318	0.997	1.744
rs4925	CC	356(68.6)	203(61.5)		1.00					
	CA	148(28.5)	110(33.3)	0.060	1.988	0.972	4.064	2.020	0.979	4.165
	AA	15(2.9)	17(5.2)	0.084	1.303	0.965	1.761	1.284	0.948	1.740
	CA + AA	163(31.4)	127(38.5)	**0**.**034**	**1**.**366**	**1**.**023**	**1**.**824**	**1**.**350**	**1**.**009**	**1**.**808**
GSTO2/rs156697	AA	284(54.9)	157(48.2)		1.00					
	AG	200(38.7)	136(41.7)	**0**.**026**	**1**.**809**	**1**.**075**	**3**.**044**	**1**.**877**	**1**.**109**	**3**.**177**
	GG	33(6.4)	33(10.1)	0.165	1.230	0.918	1.648	1.231	0.916	1.656
	AG + GG	233(45.1)	169(51.8)	0.055	1.312	0.994	1.732	1.322	0.998	1.751
rs157077	TT	168(32.4)	104(31.4)		1.00					
	TC	259(49.9)	163(49.2)	0.569	1.124	0.752	1.680	1.115	0.743	1.673
	CC	92(17.7)	64(19.3)	0.918	1.017	0.743	1.391	0.991	0.721	1.361
	TC + CC	351(67.6)	227(68.6)	0.772	1.045	0.777	1.405	1.023	0.759	1.381
rs2297235	AA	356(68.6)	202(61.0)		1.00					
	AG	149(28.7)	112(33.8)	**0**.**041**	**2**.**140**	**1**.**033**	**4**.**432**	**2**.**161**	**1**.**035**	**4**.**513**
	GG	14(2.7)	17(5.1)	0.066	1.325	0.982	1.788	1.309	0.966	1.774
	AG + GG	163(31.4)	129(39.0)	**0**.**024**	**1**.**395**	**1**.**045**	**1**.**861**	**1**.**382**	**1**.**031**	**1**.**851**
PNP/rs1713420	AA	408(78.6)	256(77.3)		1.00					
	AG	104(20.0)	71(21.5)	0.882	0.911	0.264	3.142	0.869	0.249	3.034
	GG	7(1.3)	4(1.2)	0.626	1.088	0.775	1.528	1.113	0.789	1.569
	AG + GG	111(21.4)	75(22.7)	0.662	1.077	0.773	1.501	1.097	0.784	1.534
rs1760940	AA	411(79.2)	252(76.1)		1.00					
	AC	100(19.3)	73(22.1)	0.712	1.223	0.420	3.566	1.232	0.418	3.631
	CC	8(1.5)	6(1.8)	0.315	1.191	0.847	1.673	1.193	0.846	1.682
	AC + CC	108(20.8)	79(23.9)	0.294	1.193	0.858	1.659	1.196	0.857	1.668
rs3790064	AA	433(83.8)	251(77.0)		1.00					
	AG	77(14.9)	66(10.2)	0.118	2.218	0.816	6.028	2.073	0.756	5.679
	GG	7(1.4)	9(2.8)	**0**.**035**	**1**.**479**	**1**.**028**	**2**.**127**	**1**.**468**	**1**.**018**	**2**.**118**
	AG + GG	84(16.2)	75(23.0)	**0**.**015**	**1**.**540**	**1**.**088**	**2**.**181**	**1**.**520**	**1**.**070**	**2**.**158**
Years of cigarette smoking	0	255(77.0)	382(73.6)		1.00					
	1–35	48(14.5)	109(21.0)	**0**.**030**	**1**.**516**	**1**.**042**	**2**.**205**	**1**.**735**	**1**.**152**	**2**.**611**
	≥35	28(8.5)	28(5.4)	0.148	1.498	0.867	2.589	1.209	0.660	2.215

*Logistic regression adjusted for age, gender, smoking status, drinking status and ethnicity.

### Stratification analyses of AS3MT, GSTO1, GSTO2 and PNP polymorphisms and risk of arsenic-included skin lesions

We further evaluated the influence of genotypes on arsenic-induced skin lesion risk after stratifying participants by sex, age, years of cigarette smoking and drinking (Table 3). The analysis stratified by sex revealed an evident increased risk of arsenic-induced skin lesions among female subjects carrying at least one G allele for rs3790064 of PNP (OR = 1.63, 95% CI = 1.01–2.61). When stratified by age (<55 or ≥ 55 years old), older subjects (≥55 years old) carrying at least one C allele (TC + CC) or the CC genotype for rs11191979, at least one A allele (GA + AA) or the AA genotype for rs2164624 or at least one A allele (CA + AA) or the AA genotype for rs4925, the AG genotype or at least one G allele for rs156697, the AG genotype or at least one G allele for rs2297235 and the GG genotype or at least one G allele for rs3790064 had a higher susceptibility to skin lesions than homozygous wild-type individuals. When stratified by years of cigarette smoking (0, 1–35 or >35 years), subjects smoking for 1–35 years and carrying the GG genotype or at least one G allele for the rs3790064 polymorphism had a higher susceptibility to skin lesions than homozygous wild-type individuals.

**Table 3:** Stratification analyses between polymorphisms and arsenic-induced skin lesions risk by sex, age, years of cigarette smoking.

SNP	genotype	sex OR(95% CI)		age OR(95% CI)		years of cigarette smoking OR(95% CI)		
		male	female	≥55	<55	0	1–35	>35
rs11191979	TT	1	1	1	1	1	1	1
	TC	2.40(0.66,8.71)	1.92(0.81,4.48)	2.52(0.85,7.41)	1.57(0.60,4.10)	1.80(0.82,3.94)	7.97(0.79,79.43)	0
	CC	1.31(0.83,2.05)	1.37(0.91,2.05)	**2**.**14(1**.**38**,**3**.**32)**	0.85(0.56,1.29)	1.30(0.92,1.82)	1.50(0.6,3.31)	0.94(0.30,2.92)
	TC + CC	1.37(0.89,2.13)	1.43(0.97,2.10)	**2**.**18(1**.**43**,**3**.**32)**	0.91(0.61,1.36)	1.35(0.97,1.87)	1.77(0.83,3.75)	0.85(0.28,2.58)
rs2164624	GG	1	1	1	1	1	1	1
	GA	1.57(0.44,5.56)	1.43(0.48,4.25)	0.87(0.22,3.36)	2.12(0.67,6.64)	1.86(0.74,4.68)	0.80(0.08,8.00)	0
	AA	1.31(0.83,2.08)	1.45(0.96,2.18)	**1**.**90(1**.**21**,**2**.**98)**	1.01(0.66,1.54)	1.34(0.94,1.91)	1.36(0.62,3.00)	1.29(0.43,3.89)
	GA + AA	1.33(0.85,2.08)	1.45(0.97,2.15)	**1**.**78(1**.**15**,**2**.**75)**	1.08(0.72,1.63)	1.38(0.99,1.95)	1.29(0,61,2,78)	1.16(0.39,3.44)
rs4925	CC	1	1	1	1	1	1	1
	CA	2.38(0.66,8.66)	1.91(0.80,4.56)	2.49(0.84,7.31)	1.57(0.60,4.10)	1.81(0.82,3.95)	7.63(0.76,76.07)	0
	AA	1.28(0.82,2.01)	1.33(0.89,2.00)	**2**.**04(1**.**31**,**3**.**17)**	0.85(0.56,1.29)	1.30(0.92,1.83)	1.27(0.57,2.84)	0.94(0.30,2.92)
	CA + AA	1,34(0.87,2.08)	1.40(0.95,2.06)	**2**.**08(1**.**36**,**3**.**18)**	0.92(0.61,1.36)	1.35(0.97,1.88)	1.53(0.72,3,25)	0.85(0.28,2.58)
rs156697	AA	1	1	1	1	1	1	1
	AG	1.84(0.82,4.13)	1.80(0.91,3.57)	**3**.**79(1**.**73**,**8**.**33)**	0.94(0.46,1.91)	1.72(0.95,3.10)	3.35(0.83,13.48)	0.61(0.08,4.34)
	GG	1.34(0.87,2.08)	1.16(0.78,1.73)	1.47(0.96,2.26)	1.03(0.68,1.54)	1.23(0.88,1.72)	1.08(0.51,2.31)	1.00(0.33,3.03)
	AG + GG	1.41(0.93,2.14)	1.25(0.86,1.82)	**1**.**72(1**.**14**,**2**.**58)**	1.01(0.69,1.48)	1.30(0.94,1.79)	1.31(0.64,2.64)	0.92(0.31,2.67)
rs2297235	AA	1	1	1	1	1	1	1
	AG	2.40(0.66,8.71)	2.11(0.87,5.14)	2.94(0.96,9.01)	1.57(0.60,4.10)	1.95(0.88,4.33)	7.96(0.79,79.43)	0
	GG	1.31(0.83,2.05)	1.35(0.90,2.03)	**2**.**11(1**.**36**,**3**.**26)**	0.85(0.62,1.29)	1.28(0.91,1.81)	1.50(0.68,3.31)	0.94(0.30,2.92)
	AG + GG	1.37(0.89,2.13)	1.43(0.97,2.10)	**2**.**18(1**.**43**,**3**.**32)**	0.92(0.62,1.36)	1.35(0.97,1.87)	1,77(0.83,3.75)	0.85(0.28,2.58)
rs379064	AA	1	1	1	1	1	1	1
	AG	0.93(0.22,3.99)	5.61(1.12,28.20)	2.17(0.30,15.64)	1.93(0.60,6.21)	2.25(0.70,7.21)	2.82(0.17,46.39)	1.00(0.05,17.06)
	GG	1.51(0.88,2.59)	1.44(0.88,2.37)	**1**.**77(1**.**05**,**3**.**00)**	1.24(0.75,2.05)	1.29(0.85,1.96)	**2**.**82(1**.**12**,**7**.**10)**	1.20(0.31,4.54)
	AG + GG	1.44(0.86,2.41)	**1**.**63(1**.**01**,**2**.**61)**	**1**.**79(1**.**07**,**2**.**99)**	1.32(0.82,2.12)	1.36(0.91,2.03)	**2**.**82(1**.**16**,**6**.**88)**	1.16(0.33,4.06)

### Analysis of haplotype association with arsenic-induced skin lesions risk

**Figure 1:**
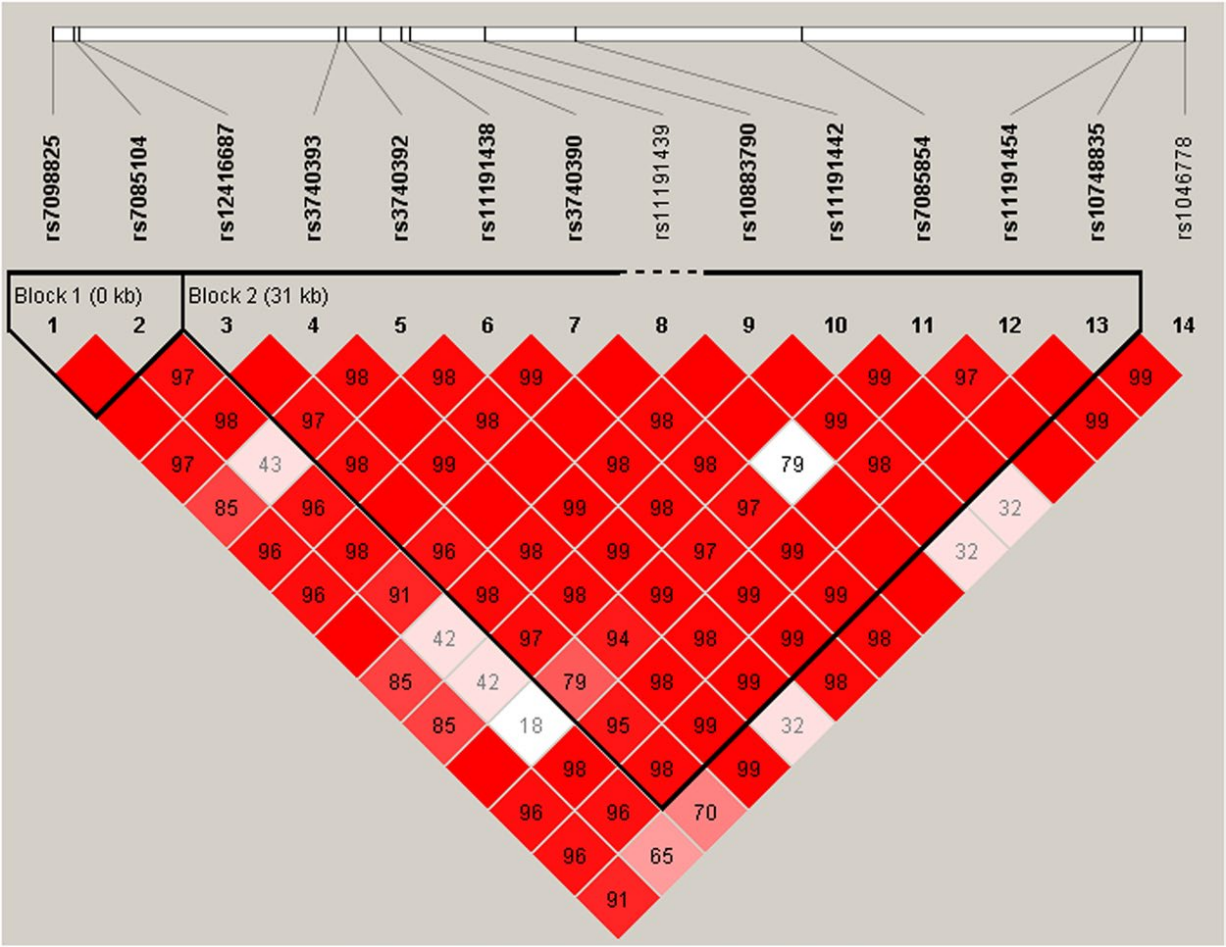
LD patterns and haplotype blocks of cases and controls were defined according to the ‘spine of LD’, as based on each end marker of a block having a D’ value > 0.8. A standard color scheme is used to display the LD pattern, with black for perfect LD (r^2^ = 1), white for no LD (r^2^ = 0) and shades of gray for intermediate LD (0 < r^2^ < 1).

Linkage disequilibrium (LD) patterns among AS3MT polymorphisms in cases and controls were investigated to determine haplotype blocks in the study population. Pairwise LD (D′) values between SNPs are indicated by the graphical overview of LD structure shown in Fig. 1. All 13 SNPs were split into two identified haplotype blocks. Overall distributions of haplotypes were not significantly different between the cases and controls (P > 0.05). Adjusted P values for haplotype blocks were also confirmed by the permutation test (Table 4), which revealed no significant differences. LD patterns among GSTO1 polymorphisms were also assessed, with all 5 SNPs split into two identified haplotype blocks. The distribution of haplotype CT between rs4925 and rs11191979 in the case group and the control group was statistically significant (P < 0.05), and haplotype CT appeared to confer a high risk of arsenic-included skin lesions (P = 0.030, OR = 1.377, 95% CI = 1.03–1.84). The cases and controls were also assessed for LD patterns among GSTO2 polymorphisms; the three SNPs grouped into one identified haplotype block. Statistical significance (P < 0.05) was observed for the distribution of haplotype GCG, which appeared to result in a high risk of arsenic-included skin lesions (P = 0.029, OR = 2.197, 95% CI = 1.08–4.44), in the cases and controls.

**Table 4:** Estimated AS3MT, GSTO1 and GSTO2 haplotypes in cases and controls.

Gene	Block	Haplotype	Frequency	Case/control counts	X2	P value	Permutation P value
AS3MT	Block1	TA	0.536	355.0:307.0/557.0:481.0	0.000	0.9886	1.0000
		CG	0.273	181.9:480.1/282.8:755.2	0.012	0.9127	1.0000
		TG	0.19	125.1:536.9/198.2:839.8	0.011	0.9154	1.0000
	Block2	TGTGCATTAG	0.455	296.0:354.0/469.0:558.0	0.003	0.9589	1.0000
		TCTCTATTGA	0.261	172.0:478.0/267.0:760.0	0.044	0.8338	1.0000
		CGCCCCACAA	0.101	63.1:586.9/107.3:919.7	0.242	0.6228	1.0000
		TGCCCCATAA	0.081	52.1:597.9/84.3:942.7	0.021	0.8852	1.0000
		TGCCCCACAA	0.078	50.9:599.1/79.7:947.3	0.003	0.9554	1.0000
		CGCCCCATAA	0.019	15.9:634.1/15.7:1011.3	1.834	0.1757	0.8435
GSTO1	block1	GA	0.727	462.0:200.0/772.0:264.0	4.548	0.0330	0.1047
		AC	0.168	128.8:533.2/157.0:879.0	5.357	0.0206	0.0694
		GC	0.105	71.2:590.8/107.0:929.0	0.076	0.7831	1.0000
	block2	CT	0.808	515.0:147.0/859.0:179.0	6.419	0.0113	**0**.**0365**
		AC	0.188	143.0:519.0/177.0:861.0	5.474	0.0193	0.0671
GSTO2	block1	ATA	0.565	368.7:293.3/592.5:445.5	0.316	0.5740	0.9341
		GCG	0.189	145.9:516.1/174.9:863.1	7.127	0.0076	**0**.**0200**
		ACA	0.158	90.8:571.2/177.0:861.0	3.382	0.0659	0.1424
		ACG	0.084	54.2:607.8/89.0:949.0	0.080	0.7773	1.0000

### Relationship between enzymatic activity and polymorphism

Enzyme-linked immunosorbent assay (ELISA) was used to determine the enzymatic activity of GSTO1, GSTO2 and PNP, as presented in Table 5. Compared with homozygous wild-type individuals, subjects who carried at least one mutant allele for GSTO1 SNPs rs11191979, rs2164624, rs2282326 and rs4925 exhibited a significant decrease in GSTO1 enzymatic activity (P < 0.05); in addition, participants carrying at least one mutant allele for GSTO2 SNPs rs156697, rs157077 and rs2297235 showed significant decreases GSTO2 activity (P < 0.05), and those carrying at least one mutant allele for PNP SNPs rs1760940, rs1713420 and rs3790064 showed significant decreases in PNP (P < 0.05).

**Table 5:** Relationship between enzyme activity and polymorphism.

Gene	Gene loci	Genotype	n	Mean	t	P	Gene	Gene locus	Genotype	n	Mean	t	P
GSTO1	rs11191979	TT	558	3.185 ± 0.987	4.303	<0.05	GSTO2	rs157077	TT	272	3.437 ± 0.168	5.004	<0.05
		TC + CC	292	2.870 ± 1.067					TC + CC	578	3.082 ± 0.102		
	rs2164624	GG	585	3.156 ± 1.013	3.385	<0.05		rs2297235	AA	537	3.200 ± 0.182	3.960	<0.05
		GA + AA	262	2.880 ± 1.034					AG + GG	273	2.891 ± 0.113		
	rs2282326	AA	451	3.324 ± 1.007	5.065	<0.05	PNP	rs1713420	AA	631	5.649 ± 0.275	5.434	<0.05
		AC + CC	399	2.890 ± 1.015					AG + GG	179	5.024 ± 0.137		
	rs4925	CC	559	3.195 ± 0.983	4.732	<0.05		rs1760940	AA	632	5.624 ± 0.276	5.317	<0.05
		CA + AA	290	2.848 ± 1.070					AC + CC	178	5.132 ± 0.137		
GSTO2	rs156697	AA	426	3.450 ± 0.124	4.518	<0.05		rs3790064	AA	652	5.445 ± 0.131	4.407	<0.05
		AG + GG	377	3.157 ± 0.127					AG + GG	151	4.927 ± 0.243		

## Discussion

Genetic polymorphisms affect the risk of certain diseases and alter individual susceptibility to disease. Indeed, epidemiological studies have confirmed that there is a great difference in susceptibility to arsenic exposure in people in the same area due to differences in arsenic metabolism in various populations^27^. For instance, the incidence of skin lesions, bladder cancer and lung cancer was found to be higher in those with arsenic poisoning in northern Chile than in Taiwan, Mexico and India^28^. Although high-arsenic water is consumed in many countries and regions, only a fraction of the exposed individuals develop skin lesions or other arsenic-induced diseases. This heterogeneity may be caused by genetic differences^23,29,30^. In this case-control study in an arsenic-exposed population in northwest China, we found a correlation between arsenic-induced skin lesions and polymorphisms of arsenic-associated metabolic enzyme genes. The results showed that polymorphisms in GSTO1 SNPs rs11191979, rs2164624, rs2282326 and rs4925, GSTO2 SNPs rs156697 and rs2297235, and PNP SNP rs3790064 affect risk to arsenic-induced skin lesions. In addition, Hui Shen *et al*. reported that some chemicals in cigarettes can influence the enzymes involved in methylation processes, especially those involved in the second methylation phase. Moreover, smoking itself may be a pathway for arsenic exposure if the cigarettes contain trace amounts of arsenic^31^. In this study, we found that those who smoked for 1–35 years were more susceptible to arsenic-induced skin lesions than non-smokers. Although related gene polymorphisms and smoking interact in skin carcinogenesis due to arseniasis, the specific mechanisms by which smoking leads to changes remain poorly understood. Previous studies have shown that effective control of smoking is an important measure to reduce the incidence of arsenic-induced skin lesions.

### GSTO1

GSTO1 is a multifunctional enzyme that is involved in many biological processes and plays a critical role in cellular detoxification systems^32–34^. According to the classical model of arsenic metabolism^35^, GSTO1 catalyzes the rate-limiting step of arsenic biotransformation *in vivo*. Many studies have investigated the relationship between the polymorphisms rs4925 and rs156697 and susceptibility to diseases, including Alzheimer’s disease, breast cancer and bladder cancer^36–41^. A C → A transition at position 419 in GSTO1 rs4925 results in alteration of the 140th amino acid from alanine (Ala) to aspartic acid (Asp). This change decreases the enzymatic activity of GSTO1 and thus affects arsenic metabolism, which reduces the arsenic biotransformation ability^42^. In the present study, we analyzed the relationship between GSTO1 polymorphism and its activity and found that participants who carried at least one mutant allele exhibited a significant decrease in GSTO1 enzymatic activity compared with homozygous wild-type individuals. From this perspective, such a polymorphism in GSTO1 may have an effect on susceptibility to certain diseases. The frequency of the rs4925 A allele in our population was 0.190, which was close to the reported frequency in the Chinese Han population, though great variation in this frequency has been found worldwide^43,44^. Our results suggest that the risk of arsenic-related skin lesions for subjects who carry at least one A allele for rs4925 is 1.36 times higher than in subjects who are homozygous wild-type; thus, the mutant allele (A) may be a risk factor for arsenic-induced skin lesions. This result is consistent with our previous findings in a urinary arsenic metabolism model^30^. In previous studies, the %DMA of individuals with the rs4925 CA genotype of GSTO1 was significantly reduced compared with that of individuals with the CC genotype. This finding also shows that the A allele is a risk factor for arsenic-induced skin lesions. To date, few studies have investigated rs2164624 and rs11191979 polymorphisms, and our results show that the AA genotype and the mutant A allele of rs2164624 and the mutant C allele of rs11191979 increase the risk of arsenic-related skin lesions. In addition, hierarchical regression indicated that the mutant C allele of rs11191979, the mutant A allele of rs2164264 and the mutant A allele of rs4925 are risk factors for arsenic-induced skin lesions in older individuals (≥55 years old). The meta-analysis by Hui Shen *et al*. indicated that older people might have poorer methylation capacity and thus be susceptible to arsenic-induced damage. In future research, we will investigate the relationship between age and urinary arsenic metabolites. For haplotype CT, the distribution of between rs4925 and rs11191979 in the case and control groups was statistically significant (P < 0.05). The frequency of the mutant A allele of rs2164624 and that of the mutant C allele of rs11191979 were 0.169 and 0.191, respectively, in our study population, values that are close to the reported 0.1897 and 0.1763 global minor allele frequency (MAF) values, respectively.

### GSTO2

Multivariate logistic regression analysis revealed that the AG genotype of rs156697 and the AG genotype and mutant G allele of rs2297235 increased the risk of arsenic-related skin lesions. Moreover, hierarchical regression indicated that the AG genotype of rs156697 and the AG genotype and mutant G allele of rs2297235 are risk factors for arsenic-induced skin lesions in older subjects (≥55 years old). GSTO2 and GSTO1 exhibit 64% amino acid sequence identity, and the dehydroascorbate reductase activity of GSTO2 is 70–100 times higher than that of GSTO1^45^. These results suggest that by recycling ascorbate, GSTO2 may play a significant role in protecting against oxidative stress. The rs156697 mutant G allele frequency was 0.28 in our study of 850 Chinese subjects, which was similar to the frequency reported in Turkish (0.219), Japanese (0.216) and Hong Kong Chinese (0.270) populations^43,46^. The A → G transition at position 424 of the exon in GSTO2 rs156697 changes an Asn to aspartic acid an Asp, and in our previous study, we reported that the mutant G allele might be a risk factor for arsenic-induced skin lesions^30^. Analysis of expression patterns showed that GSTO2 expression is low in human tissues, suggesting that GSTO2 may have a critical role in cellular signal transduction^47^. Other researchers have also studied rs156697 polymorphisms in GSTO2^43^. We found these polymorphisms to be associated with decreased enzyme activity in arsenic metabolism, consistent with previous studies^43,48^. Regarding the rs2297235 SNP, Ema G. Rodrigues *et al*. revealed that homozygous wild-type GSTO2 rs2297235 is significantly associated with higher urinary MMA and DMA concentrations in individuals from an arsenic-exposed region in Bangladesh^49^. Our research shows that GCG haplotype is a risk factor for arsenic-induced skin lesions. As the effects of haplotype on phenotype include synergistic or antagonistic effects of genes and are predominately influenced by certain genes, further analysis is needed due to such complex interactions among genes.

### PNP

The classical pathway suggests that As^V^ undergoes sequential reduction and oxidative methylation after entering the cell via a phosphate transporter. In the human liver, PNP reduces As^V^ to As^III^ ^25,50^, and many epidemiological studies have reported a positive association between PNP polymorphisms and arsenic-induced skin lesions^16,25^. For example, De *et al*. found three exonic polymorphisms of PNP (His20His, Gly51Ser and Pro57Pro) to be significantly associated with arsenism^19^. In addition, Fen Wu indicated that rs17886095, rs17882804 and rs3790064 in PNP are significantly correlated with urinary arsenic metabolites in a population exposed to high levels of arsenic in drinking water in Bangladesh^51^. It was also reported that there is no significant correlation between PNP polymorphisms and urinary arsenic metabolism^52^. In our study, we evaluated three polymorphic loci of PNP and found that SNPs rs1713420 and rs1760940 were not associated with the risk of arsenic-induced skin lesions; conversely, the mutant G allele of rs3790064 did increase the risk of arsenic-related skin lesions in our study population. We found that individuals with the GG genotype had increased odds (1.473) of skin lesions compared with individuals harboring the rs3790064 AA genotype (OR = 1.468 (95% CI, 1.018–2.118)); individuals with the mutant G allele of rs3790064 also displayed increased odds (1.520) of skin lesions compared with individuals harboring the rs3790064 AA genotype (OR = 1.520 (95% CI, 1.070–2.158)). Moreover, hierarchical regression indicated that the mutant G allele of rs3790064 is risk factor for arsenic-induced skin lesions in those who have smoked for 1-35 years, with these individuals being more susceptible to arsenic-induced skin lesions than non-smokers. The mutant G allele of SNP rs3790064 is risk factor for arsenic-induced skin lesions in older subjects (≥55 years old) and females. However, Haploview analysis did not identify a block with significant LD among the nine SNPs (rs1713420, rs1760940, rs3790064). The association between PNP polymorphisms and arsenic metabolism is controversial^52,53^. Therefore, further studies should consistently explore the function of PNP in arsenic metabolism and the relationship between PNP polymorphisms and arsenic-induced skin lesions using a larger arsenic-exposed population.

### AS3MT

AS3MT, located in the 10q24.32 region of chromosome 10, encodes the main methyltransferase involved in arsenic metabolism and affects an individual’s efficiency to detoxify ingested arsenic^54^. Gordon Gong *et al*. discovered that the risk of coronary heart disease and hyperlipidemia was higher for subjects with rs10748835 polymorphism who carry the AG genotype than for subjects who carry the AA genotype^55^. Several *in vitro* and *in vivo* studies have revealed that AS3MT is essential for oxidative methylation of trivalent arsenic during the arsenic biotransformation process, which highlights the importance of the AS3MT enzyme in converting inorganic arsenic metabolites to their corresponding methylated products^20,56^. Genetic variations in AS3MT can lead to individual differences in inorganic arsenic metabolites, which is one of the main reasons for the different susceptibilities to endemic arsenic poisoning within a population exposed to high arsenic levels^57^. Regardless, the results of this study showed that the 13 polymorphic loci of a intron in AS3MT (rs1046778, rs10748835, rs10883790, rs11191438, rs11191442, rs11191454, rs12416687, rs3740390, rs3740392, rs3740393, rs7085104, rs7085854 and rs7098825) were not associated with arsenic-induced skin lesions; overall distributions of haplotypes were not significantly different between cases and controls (P > 0.05). In a previous study^30^, we investigated the relationship between rs10748835 (intron10-A35991G) polymorphism and urinary arsenic metabolism and found that this locus was not associated with the risk of endemic arsenism from contaminated drinking water. Although several studies have shown that AS3MT gene polymorphisms do not cause differences in disease susceptibility^21,36,58^, we believe that this result does not reflect the significance of the AS3MT gene in arsenic metabolism, possibly due to differences in populations and races.

In conclusion, the three typical arsenism areas in China were selected for study, and the samples were representative. The variants of GSTO1, GSTO2 and PNP render the susceptible toward developing arsenic-induced skin lesions in individuals exposed to high-dose inorganic arsenic in northwest China. We did not find significant association of 13 SNPs at AS3MT locus with arsenic-induced skin lesions, in future researchs, we will study the effects of the AS3MT gene on individual urinary arsenic metabolism based on urinary arsenic methylation metabolism levels in populations exposed to high arsenic levels.

### Limitation

One limitation of our study is that we did not investigate the association between gene polymorphisms and urinary arsenic speciation. However, we suggest that a larger sample size is needed, owing to the various metabolites involved.

## Materials and Methods

### Study sites and subject selection

According to the Endemic Disease Control Center of the Chinese Center for Disease Control and Prevention and previous knowledge of the situation in areas with endemic arsenism in China, three adjacent districts were selected in northwest China (Gansu Province, Shanxi Province and the Inner Mongolia Autonomous Region). The three provinces are typical arsenism areas in China. Previous research has described the epidemiological investigation and sample collection in Gansu Province in detail^30^. Hanggin Rear Banner of the Bayannur League in the Inner Mongolia Autonomous Region, Tianzhen County in Shanxi Province, and Tuoketuo County were selected as study areas; water screening for the presence of high levels of arsenic was conducted in these areas in 2005 by a national program, and a compete list of arsenic exposure was generated. In the above regions, the arsenic levels were 0–0.5102 mg/L in hand-pumped well water and 0.1670 mg/L in central well water. In total, 1412 subjects were investigated, and 1124 subjects who had explicit arsenic levels were selected. Of these 1124 subjects, 1061 were exposed to high levels of arsenic in drinking water (>0.01 mg/L). SNPs were assessed in 1040 DNA samples (samples from 21 subjects for whom whole blood samples were missing were excluded). The epidemiological survey and sample collection of the above two groups were described in previous articles^59,60^.

In the present study, we recruited 850 subjects for sample collection from arsenism areas in Gansu Province, Shanxi Province and the Inner Mongolia Autonomous Region. The subjects included 331 cases and 519 controls. Cases that lacked SNP detection results or did not meet the inclusion criterion were excluded. The inclusion criterion for the cases was the presence of skin lesions, which is the hallmark sign of arsenism, as diagnosed by the national standard (Standard of Diagnosis for Endemic Arsenism, WS/T-211-2001). For the controls, we recruited individuals without arsenic-induced skin lesions. All subjects had similar lifestyles, social backgrounds and eating habits. All subjects provided informed consent before the study, and all protocols in this study were approved by the Ethics Committee of Harbin Medical University. All methods were performed in accordance with relevant guidelines and regulations, and the Institutional Review Board of Harbin Medical University approved all experimental protocols.

### Sample collection

Migrant workers who had returned from the city and villagers who had experienced fever, infection or autoimmune diseases or who had recent occupational exposure to arsenic (within 1 month) or X-rays (within 6 months) were excluded from the blood sample collection. For the arsenism areas in Gansu Province, approximately 3 mL of fasting venous blood was extracted in the morning from villagers who were willing to provide blood samples; these samples were blended and collected in ethylenediaminetetraacetic acid (EDTA) anticoagulation tubes for mixing. After collection, the specimens were immediately transported to the laboratory in a −18 °C vehicle refrigerator and then stored at −80 °C. For the arsenism areas in Shanxi Province and the Inner Mongolia Autonomous Region, the blood samples were divided into two groups: for one group, the samples were centrifuged, and serum was collected as a back-up; the samples for the second group consisted of anticoagulated blood. Water samples were collected in 50-mL acid-washed tubes rinsed 3 times with water before sampling and stored at room temperature. Household drinking water samples were collected if a subject’s arsenic exposure history was not clear. Arsenic concentrations in water were determined by atomic absorption spectrophotometry (AA-6800, Shimadzu Co., Kyoto, Japan). IAs^III^, arsenate (iAs^V^), MMA^V^ and DMA^V^ in the 850 urine samples were measured using high-performance liquid chromatography (HPLC) for separation and hydride generation atomic fluorescence methods for detection. Before quantification, the urine samples were thawed naturally and centrifuged for 10 min at 12,000 rpm, and the supernatant was filtrated through a 0.45-μm membrane.

### Genotyping of SNPs

QIAamp51106 DNA Extraction Kit (Qiagen, Germany) was used to extract DNA from the 1040 whole blood samples. Sample DNA (10 ng) was amplified by polymerase chain reaction (PCR) according to the manufacturer’s recommendations, and SNP genotyping was performed using a custom-by-design 2 × 48-Plex SNPscan^TM^ Kit (Cat#:G0104; Genesky Biotechnologies, Inc., Shanghai, China). This kit was developed according to patented SNP genotyping technology by Genesky Biotechnologies Inc. based on double ligation and multiplex fluorescence PCR, as previously described^61,62^. To validate the genotyping accuracy using SNPscanTM Kit, eight of 92 SNP loci were analyzed by single-nucleotide extension using Multiplex SNaPshot Kit (Applied Biosystems Inc., Foster City, CA, USA) for 48 samples, with greater than 99% concordance rates. SNP genotyping was performed using the TaqMan SNP genotyping assay (Applied Biosystems Inc). The genotyping success rates were more than 99% in Stage II samples, and the concordance rates were more than 99% based on 5% duplicate samples.

### Enzyme Linked Immunosorbent Assay (ELISA)

In every sample, GSTO1, GSTO2, and PNP activities were detected using commercial enzyme-linked immunosorbent assay (ELISA) kits (Kete Biological Technology Co., Ltd, Jiangsu, China) following the manufacturer’s instructions. Briefly, 50 μl of standard solutions or DNA samples was added to 96-well plates and incubated for 30 min at 37 °C. The samples were washed 5 times, and the conjugate reagent was added and incubated for 30 min at 37 °C.The samples were again washed 5 times, and chromogen solutions A and B were added and incubated for 10 min at 37 °C. Absorbance at 450 nm was determined using a Microplate Reader (BioTek, USA).

### Statistical analysis

The statistical analysis was performed using SPSS (version 13.01 S; Beijing Stats Data Mining Co. Ltd.). We performed an independent t-test to calculate significant differences in age between the cases and controls. Differences in the distribution of categorical data (gender, smoking status, drinking status and ethnicity) were tested using the Chi-square test. The genotype and allelic gene frequencies of each site in GSTO1, GSTO2, AS3MT and PNP were calculated, and HWE was assessed in the controls using the Chi-square test. We used bivariate unconditional logistic regression analysis to estimate odds ratios (ORs) and 95% confidence intervals (CIs) between the cases and controls for the skin lesion risk with GSTO1, GSTO2, AS3MT and PNP gene polymorphisms. Multivariate unconditional logistic regression analysis with adjustment for age, gender, smoking status, drinking status and ethnicity was performed to calculate adjusted ORs and 95% CIs. The total concentration of the four different forms of arsenic (iAs^III^ + iAs^V^ + MMA^V^ + DMA^V^) was considered as the approximate total arsenic (tAs) value. Normality and homogeneity tests of variance were performed for all urinary arsenic determination indices; indices satisfying the described conditions are indicated with*, which represents the indices for the parameter test. The t-test was used for cases and controls, with a t-statistics value. The statistical tests were two-tailed probability tests with a test level of α = 0.05, and P < 0.05 was considered significant.

LD analysis and haplotype reconstruction was performed using Haploview 4.2. Common haplotypes with frequencies of >0.01 were compared between cases and controls. The P value was adjusted by a permutation test.

## References

[1] Sanchez TR, Perzanowski M, Graziano JH (2016). Inorganic arsenic and respiratory health, from early life exposure to sex-specific effects: A systematic review. Environmental research.

[2] A M, M. K S, M. A H (2006). Arsenic contamination in groundwater: a global perspective with emphasis on the Asian scenario. J Health Popul Nutr.

[3] Paul S, Majumdar S, Giri AK (2015). Genetic susceptibility to arsenic-induced skin lesions and health effects: a review. Genes and environment: the official journal of the Japanese Environmental Mutagen Society.

[4] Rodriguez-lado L (2013). Groundwater Arsenic Contamination throughout China. science translational medicine.

[5] Kim YJ, Kim JM (2015). Arsenic Toxicity in Male Reproduction and Development. Development & reproduction.

[6] Steinmaus C (2014). Elevated lung cancer in younger adults and low concentrations of arsenic in water. American journal of epidemiology.

[7] Gao J, Yu J, Yang L (2011). Urinary arsenic metabolites of subjects exposed to elevated arsenic present in coal in Shaanxi Province, China. International journal of environmental research and public health.

[8] Calatayud M, Gimeno-Alcaniz JV, Velez D, Devesa V (2014). Trivalent arsenic species induce changes in expression and levels of proinflammatory cytokines in intestinal epithelial cells. Toxicology letters.

[9] Hsu LI (2015). Association of Environmental Arsenic Exposure, Genetic Polymorphisms of Susceptible Genes, and Skin Cancers in Taiwan. BioMed research international.

[10] Ghosh P (2007). Increased chromosome aberration frequencies in the Bowen’s patients compared to non-cancerous skin lesions individuals exposed to arsenic. Mutation Research/Genetic Toxicology and Environmental Mutagenesis.

[11] Yamaoka H (2011). Multiple Bowen’s disease in a patient with a history of possible arsenic exposure:a case report. Tokai J Exp Clin Med.

[12] Sattar A (2016). Metabolism and toxicity of arsenicals in mammals. Environmental toxicology and pharmacology.

[13] Argos M (2011). A prospective study of arsenic exposure from drinking water and incidence of skin lesions in Bangladesh. American journal of epidemiology.

[14] A H, R M (2008). Genetic variations associated with interindividual sensitivity in the response to arsenic exposure. Pharmacogenomics.

[15] Das N, Giri A, Chakraborty S, Bhattacharjee P (2016). Association of single nucleotide polymorphism with arsenic-induced skin lesions and genetic damage in exposed population of West Bengal, India. Mutation research.

[16] Antonelli R, Shao K, Thomas DJ, Sams R, Cowden J (2014). AS3MT, GSTO, and PNP polymorphisms: impact on arsenic methylation and implications for disease susceptibility. Environmental research.

[17] Faita F, Cori L, Bianchi F, Andreassi MG (2013). Arsenic-induced genotoxicity and genetic susceptibility to arsenic-related pathologies. International journal of environmental research and public health.

[18] Pierce BL (2012). Genome-wide association study identifies chromosome 10q24.32 variants associated with arsenic metabolism and toxicity phenotypes in Bangladesh. PLoS genetics.

[19] De Chaudhuri S (2008). Genetic variants associated with arsenic susceptibility: study of purine nucleoside phosphorylase, arsenic (+3) methyltransferase, and glutathione S-transferase omega genes. Environmental health perspectives.

[20] Schlawicke Engstrom K (2007). Genetic polymorphisms influencing arsenic metabolism: evidence from Argentina. Environmental health perspectives.

[21] Agusa T, Fujihara J, Takeshita H, Iwata H (2011). Individual variations in inorganic arsenic metabolism associated with AS3MT genetic polymorphisms. International journal of molecular sciences.

[22] Concha G, Vogler G, Nermell B, Vahter M (2002). Intra-individual variation in the metabolism of inorganic arsenic. Int Arch Occup Environ Health.

[23] Recio-Vega R (2016). Association between polymorphisms in arsenic metabolism genes and urinary arsenic methylation profiles in girls and boys chronically exposed to arsenic. Environmental and molecular mutagenesis.

[24] Paiva L (2010). Association between GSTO2 polymorphism and the urinary arsenic profile in copper industry workers. Environmental research.

[25] Gaxiola-Robles R, Labrada-Martagon V, Bitzer-Quintero OK, Zenteno-Savin T, Mendez-Rodriguez LC (2015). Purine nucleoside phosphorylase and the enzymatic antioxidant defense system in breast milk from women with different levels of arsenic exposure. Nutricion hospitalaria.

[26] Canduri F (2004). Structures of human purine nucleoside phosphorylase complexed with inosine and ddI. Biochemical and Biophysical Research Communications.

[27] Ahsan H (2003). Susceptibility to arsenic-induced hyperkeratosis and oxidative stress genes myeloperoxidase and catalase. Cancer Letters.

[28] Smith, A. H., H.-R. C. & Bates, M. N. Cancer risks from arsenic in drinking water. *Environ Health Perspect*, 259–267 (1992). 10.1289/ehp.9297259 PMC1519547 1396465

[29] Borghini A (2016). Arsenic exposure, genetic susceptibility and leukocyte telomere length in an Italian young adult population. Mutagenesis.

[30] Fu S (2014). Urinary arsenic metabolism in a Western Chinese population exposed to high-dose inorganic arsenic in drinking water: influence of ethnicity and genetic polymorphisms. Toxicology and applied pharmacology.

[31] Shen H (2016). Factors Affecting Arsenic Methylation in Arsenic-Exposed Humans: A Systematic Review and Meta-Analysis. International journal of environmental research and public health.

[32] Josephy PD (2010). Genetic variations in human glutathione transferase enzymes: significance for pharmacology and toxicology. Human genomics and proteomics: HGP.

[33] Hayes JD, Flanagan JU, Jowsey IR (2005). Glutathione transferases. Annual review of pharmacology and toxicology.

[34] Xu YT (2014). Genetic polymorphisms in Glutathione S-transferase Omega (GSTO) and cancer risk: a meta-analysis of 20 studies. Scientific reports.

[35] Hernández, A. & Marcos, R. Genetic variations associated with interindividual sensitivity in the response to arsenic exposure. **9**, 1113–1132 (2008). 10.2217/14622416.9.8.1113 18681785

[36] Beebe-Dimmer JL (2012). Genetic variation in glutathione S-transferase omega-1, arsenic methyltransferase and methylene-tetrahydrofolate reductase, arsenic exposure and bladder cancer: a case-control study. Environmental health: a global access science source.

[37] Allen M (2012). Glutathione S-transferase omega genes in Alzheimer and Parkinson disease risk, age-at-diagnosis and brain gene expression: an association study with mechanistic implications. Molecular neurodegeneration.

[38] Djukic T (2015). GSTO1*C/GSTO2*G haplotype is associated with risk of transitional cell carcinoma of urinary bladder. International urology and nephrology.

[39] Andonova IE (2010). No evidence for glutathione S-transferases GSTA2, GSTM2, GSTO1, GSTO2, and GSTZ1 in breast cancer risk. Breast cancer research and treatment.

[40] Li YW, Martin ER, Li YJ (2008). EMK: a novel program for family-based allelic and genotypic association tests on quantitative traits. Annals of human genetics.

[41] Lesseur C (2012). A case-control study of polymorphisms in xenobiotic and arsenic metabolism genes and arsenic-related bladder cancer in New Hampshire. Toxicology letters.

[42] Deng X (2015). GSTP1 and GSTO1 single nucleotide polymorphisms and the response of bladder cancer patients to intravesical chemotherapy. Scientific reports.

[43] Whitbread AK, Tetlow N, Eyre HJ, Sutherland GR, Board PG (2003). Characterization of the human Omega class glutathione transferase genes and associated polymorphisms. Pharmacogenetics.

[44] Hirakawa M, T. T. & Hasimoto, Y. JSNP_ a database of common gene variations in the Japanese popμlation.pdf. *Nucleic Acid Res*, 158–162 (2002). 10.1093/nar/30.1.158 PMC99126 11752280

[45] Xu Y (2009). Lack of association of glutathione-S-transferase omega 1(A140D) and omega 2 (N142D) gene polymorphisms with urinary arsenic profile and oxidative stress status in arsenic-exposed population. Mutation research.

[46] Takeshita H (2009). Diversity of glutathione s-transferase omega 1 (a140d) and 2 (n142d) gene polymorphisms in worldwide populations. Clinical and experimental pharmacology & physiology.

[47] Rossman TG, Uddin AN, Burns FJ (2004). Evidence that arsenite acts as a cocarcinogen in skin cancer. Toxicol Appl Pharmacol.

[48] Zakharyan, R. A., A.-F. F. & Cμllen, W. R. Enzymatic methylation of arsenic compounds. VII. Monomethylarsonous acid (MMAIII) is the substrate for MMA methyltransferase of rabbit liver and human hepatocytes. *Toxicol Appl Pharmacol* **158**, 9 (1999). 10.1006/taap.1999.8687 10387927

[49] Rodrigues EG (2012). GSTO and AS3MT genetic polymorphisms and differences in urinary arsenic concentrations among residents in Bangladesh. Biomarkers: biochemical indicators of exposure, response, and susceptibility to chemicals.

[50] Jomova K (2011). Arsenic: toxicity, oxidative stress and human disease. Journal of applied toxicology: JAT.

[51] Wu F (2014). Interaction between arsenic exposure from drinking water and genetic susceptibility in carotid intima-media thickness in Bangladesh. Toxicology and applied pharmacology.

[52] Meza MM (2005). Developmentally Restricted Genetic Determinants of Human Arsenic Metabolism: Association between Urinary Methylated Arsenic and CYT19 Polymorphisms in Children. Environmental health perspectives.

[53] Nemeti B, Gregus Z (2004). Glutathione-dependent reduction of arsenate in human erythrocytes–a process independent of purine nucleoside phosphorylase. Toxicol Sci.

[54] Mazumdar M (2015). Polymorphisms in maternal folate pathway genes interact with arsenic in drinking water to influence risk of myelomeningocele. *Birth defects research*. Part A, Clinical and molecular teratology.

[55] Gong G, O’Bryant SE (2012). Low-level arsenic exposure, AS3MT gene polymorphism and cardiovascular diseases in rural Texas counties. Environmental research.

[56] Hsueh YM (2016). Association of Arsenic Methylation Capacity with Developmental Delays and Health Status in Children: A Prospective Case-ControlTrial. Scientific reports.

[57] Drobna Z,WS, Devesa V (2005). Metabolism and toxicity of arsenic in human urothelial cells expressing rat arsenic (+3 oxidation state)-methyltransferase. Toxicol Appl Pharmacol.

[58] Fujihara J (2011). Genetic variants associated with arsenic metabolism within human arsenic (+3 oxidation state) methyltransferase show wide variation across multiple populations. Archives of toxicology.

[59] Zhao L (2010). Serum proteomic profiling analysis of chronic arsenic exposure by using SELDI-TOF-MS technology. Toxicology letters.

[60] Li Y (2012). Changes in serum thioredoxin among individuals chronically exposed to arsenic in drinking water. Toxicology and applied pharmacology.

[61] Chen X (2012). Genome-wide association study validation identifies novel loci for atherosclerotic cardiovascular disease. Journal of thrombosis and haemostasis: JTH.

[62] Yang XL-NW (2015). B-cell Iymphoma2 rs17757541C > G polymorphism was associated with an increased risk of coronary artery disease in a Chinese population. Int J Clin Exp Pathol.

